# Making Video Games More Inclusive for People Living With Motor Neuron Disease: Scoping Review

**DOI:** 10.2196/58828

**Published:** 2024-12-23

**Authors:** Ben O'Mara, Matthew Harrison, Kirsten Harley, Natasha Dwyer

**Affiliations:** 1 Department of Media & Communication Faculty of Health, Arts & Design Swinburne University Melbourne Australia; 2 Centre for Social Impact University of New South Wales Sydney Australia; 3 Melbourne Graduate School of Education University of Melbourne Melbourne Australia; 4 Centre for Disability Research and Policy University of Sydney Sydney Australia; 5 College of Arts, Business, Law, Education and IT Victoria University Footscray Park Australia

**Keywords:** motor neuron disease, video games, inclusion, information technology, well-being, disability, mobile phone

## Abstract

**Background:**

Evidence suggests that individuals with motor neuron disease (MND), a terminal illness, find enjoyment and social connection through video games. However, MND-related barriers can make gaming challenging, exacerbating feelings of boredom, stress, isolation, and loss of control over daily life.

**Objective:**

We scoped the evidence to describe relevant research and practice regarding what may help reduce difficulties for people with MND when playing video games.

**Methods:**

A scoping review was conducted using the Arksey and O’Malley framework, recent scoping review guidance, and engaging with people with lived experience of MND. Peer-reviewed studies were sourced from PubMed and the Swinburne University of Technology Library. Gray literature was identified from government, not-for-profit, commercial, and community websites. Data were extracted and summarized from the collected literature.

**Results:**

The evidence base, consisting of quantitative and qualitative research, lived experience stories, information resources, reviews, and guidelines, included 85 documents. Only 8 (9%) directly addressed video games and people with MND; however, these studies were limited in depth and quality. The primary technologies examined included customized information and communications technology for communication and control of computing systems (including desktop, laptop, smartphone, tablet, and console systems) and video game software and hardware (including hand controllers and accessibility features, such as difficulty level, speed, and remappable buttons and controls). Common factors in the evidence base highlight barriers and enablers to enjoying video games for people with MND. These include technological, physical, social, and economic challenges. Addressing these requires greater involvement of people with MND in game and technology research and development. Changes to information and communications technology, game software and hardware, policies, and guidelines are needed to better meet their needs.

**Conclusions:**

There is a significant gap in understanding the lived experience of people with MND with video games and what makes playing them easier, including appropriate customization of technology and the social experience of games. This gap perpetuates exclusion from gaming communities and recreation, potentially worsening social isolation. Existing evidence suggesting viable options for future research and practice. Video game and information and communications technology research and development must prioritize qualitative and quantitative research with people with MND at an appropriate scale, with a focus on lived experience, use of improved participant engagement techniques, and user-focused design for more inclusive games. Practical work needs to increase awareness of what can help make games more inclusive, including incorporation of accessibility early in the game production process, early incorporation of accessibility in game production, and affordable options for customized interfaces and other devices to play games.

## Introduction

### Background

While there is an extensive focus on medical research for people living with motor neuron disease (MND), there is a need to more seriously explore how improved access to and use of communication technology can support greater social inclusion and quality of life for those living with this condition [1]. MND is debilitating and causes progressive muscle weakness and disability that can severely limit the ability to maintain social connections; a sense of community; and participation in enjoyable activities as part of daily life, including activities such as playing video games [2-6]. For many people with MND, increasing difficulties socializing and spending time with loved ones or simply using a computer to play a favorite game may lead to feelings of apathy, depression, and a lack of respect [7]. As a result, quality of life can deteriorate [8-10]. More broadly, inclusive, equitable opportunities for people living with MND to enjoy playing video games, similarly to many other everyday activities, firmly aligns with the vision set out in the Convention on the Rights of Persons with Disabilities [11].

Therefore, finding ways to better support social inclusion and provide more opportunities for participation and communication is of paramount importance for people living with MND [12,13]. Technologies for engaging in recreational activities such as video games can help promote social inclusion, reduce boredom, and facilitate social connection and a sense of dignity and choice as part of daily life while living with MND [14-16]. This is not a radical contention as gaming and social activity regarding play support a sense of connection for many historically marginalized populations, helping reduce the feelings of isolation and loneliness through affording emotional support and a sense of belonging [14]. There is emerging evidence suggesting that playing and discussing video games on smartphones, computers, tablets, and consoles remains of great interest to many people living with MND despite their deteriorating physical health as they offer an important opportunity to share experiences, find comfort in community, and simply have fun [6]. However, there are significant gaps in understanding and evidence on what helps make video games more fun and easier to play for those with MND. The gaps risk worsening the experience of living with MND for those who want to play video games by making them harder to access and enjoy and limiting the ways in which future work can better respond to the needs of players with MND.

Furthermore, there are gaps in evidence despite the fact that the broader perception of video games as a trivial pursuit for children is changing and gamer culture is evolving with its own norms and values. Contrary to stereotypes, a recent survey found that 81% of all Australians play video games, with the average age of gamers being approximately 35 years [17], indicating that adults who grew up playing video games continue to be active players. Gender representation in gaming is more balanced than media portrayals suggest, with an almost equal split between male and female players [17]. Different gaming communities have their own subcultural differences but also share some commonalities. “Gamers” self-identify with gamer culture and play specific types of games known as “hardcore,” which require skill and knowledge. Mastering these games increases social and cultural capital within gaming communities.

Gamer culture extends beyond the game itself to the community forming around it, known as the “metagame.” For example, gamers also engage in “modding” or customizing games to demonstrate mastery and understanding of game design. Social capital plays a crucial role in this community, and gamers use social media platforms such as Twitch and YouTube Gaming to share their gameplay, knowledge, and critique. Live streaming has created a growing industry of streaming celebrities. In addition, fan fiction and expanded universes have emerged, where players create stories set in game worlds. Understanding gamer culture’s impact on players is vital for understanding the affordances and tensions in creating spaces for social inclusion through play.

Social experiences of games are similarly important for players with MND. Players connect with others to learn what games and devices help them keep playing after a diagnosis of MND and manage its associated challenges [18]. Online communities of players have created valuable information and opportunities to share what helps play games for those with MND [19,20]. Carers, loved ones, occupational therapists, and other health and community professionals are often important in assisting with the setup of gaming systems, helping with placement and use of technology, and problem-solving technical issues [6]. Game and technology developers work to create games that are easier to play for people with a range of mobility and movement issues [21,22], and government-funded support for access to information and communications technology can be important in being able to afford to play [9,23].

Game developers and online communities of gamers are increasingly exploring creative and customized options for improved gameplay for those with disabilities [15,16]. Campaigns focusing on games and MND and have created social opportunities and awareness of how people deal with the challenges of living with MND, which can help foster an environment that values the contributions of individuals with disabilities [9,24]. However, despite the potential benefits, there is limited focused research on the conditions and characteristics that facilitate making video games more inclusive for people living with MND.

### Objectives

To address the gap, our scoping review’s objective was to provide an overview of existing academic and gray literature evidence from multiple fields of study (eg, health, education, and disability and work that is not peer reviewed) on the experiences of people living with MND and those with related conditions with playing video games. Increased understanding of what can help make video games more inclusive when playing and as a social experience is a valuable opportunity for future work to better meet the needs of people with MND. With more fun and inclusive games, it is possible to help more people with MND maintain quality of life and a sense of dignity, qualities that many gamers and others in society may take for granted. Future work on MND and games may also benefit from insights generated at the intersection of relevant fields, including health, social inclusion, communication technology, education, and information about the lived experience of MND.

As part of our objective, we hoped to balance the academic literature and better map the available evidence by identifying relevant work in gray literature outside academia [25]. This included evaluations, information resources, and communication work retrieved from government, nonprofit, technology development, and commercial databases. By mapping what evidence is available outside the traditional confines of academic literature, our review hoped to broaden the conversation to include more voices of people living with MND. In working toward our objective, this scoping review sought to identify which areas of future research should be prioritized for making video games more inclusive for people with MND.

In this review, we use the term *MND* in a generic way and to describe a collection of diseases in which motor neurons deteriorate. The term includes amyotrophic lateral sclerosis (ALS), which is a common type of the disease [26]. We also use the term *information and communications technology* for the large variety of devices and customized interfaces that people with MND use for communication, daily activities, and enjoyment of video games. This term includes devices defined as “assistive technology,” “augmentative technology,” “communication technology,” and variations on these definitions for technology used to restore user functionality and overcome problems such as reduced physical movement and difficulty operating computers, smartphones, televisions, and keyboards. Our use of the term *information and communications technology* is intended to be broad and flexible to highlight the capacity of people with MND to use a range of devices in ways that suit their needs for enjoying video games and the differing definitions of technology used to support those with disabilities worldwide.

## Methods

### Overview

Our approach to reviewing evidence was the use of an updated version of the framework by Arksey and O’Malley [27] for conducting scoping reviews to help improve methodological clarity. Minor adaptations to the framework were made based on recommendations from Pollock et al [28], Levac et al [29], the Joanna Briggs Institute [30], and Zonneveld et al [31]. The approach was developed and described in our scoping review protocol and consisted of 5 stages [6].

### Stage 1: Identifying the Research Questions

This review sought to answer five overarching research questions:

1. What is the existing evidence base of academic and gray literature on the use of video games by people with MND and opportunities for and barriers to making them more inclusive?
2. What kinds of software, hardware aids, and information and communications technology are available to enable people living with MND to play video games?
3. What are the barriers to and opportunities for improving the use of video games by people living with MND?
4. What role do video games play for people living with MND, and what adjustments can people living with MND make to enable game playing?
5. How can future research address the evidence base, including for exploring barriers and opportunities, policy development, advocacy, education, awareness-raising activities, clinical practice, and game technology development?

### Stage 2: Identifying Relevant Academic and Gray Literature

Inclusion and exclusion criteria were used to select academic and gray literature for review. The criteria for inclusion were (1) a focus on increasing use of and access to IT for playing video games, communication, or participating in daily life by people with MND or those with similar motor impairments and mobility issues (eg, spinal injury, muscular dystrophy, and cerebral palsy); (2) use of any study design, including prototype development, pilot studies, systematic reviews and evaluation reports, or information on the experiences of people living with MND and similar conditions playing video games in work that was not peer reviewed; (3) participants aged ≥18 years unless participants used IT that could be customized for use by people with MND (eg, children and young adolescents using console controllers); and (4) work published in English between 2010 and 2024.

Amendments to the criteria were made after consultation with an academic librarian and initial literature scoping and search term development. Due to the initial results suggesting major gaps in evidence, the inclusion criteria were widened to include academic and gray literature with participants experiencing similar motor impairment and mobility issues or children or adolescents using IT that could be customized for use by people with MND. Criteria and recommendations from the study protocol [6] and conduct of scoping reviews and related forms of literature searching [28,32,33] were applied to all forms of evidence.

In total, 2 electronic databases were used for academic literature: PubMed and MEDLINE and the Swinburne University of Technology Library (including Humanities and Social Sciences Collection, SPORTDiscus, and Computers and Applied Sciences). BO, MH, KH, and ND reviewed and approved databases for use through an iterative process to refine search terms in accordance with the study protocol [6]. The search was primarily conducted between June 2021 and August 2022 and then in September 2024 for ensuring the inclusion of any more recent work to adequately gather evidence from a relatively large body of studies from different disciplines, including health, information and communications technology, and education. The search terms are shown in Textbox 1. The reference lists from the identified literature were subsequently searched for relevant work to be included in the review.

Summary of search terms used to identify relevant literature for this scoping review. Asterisk (*) indicates a wildcard character that is used to search for the listed term and terms similar to the listed term.

**Diagnostic labels**

- Motor neuron disease
- Amyotrophic lateral sclerosis
- Muscular atrophy and paralysis

**Culture**

- Gaming
- eSport
- Computer games

**Software**

- Video games
- Internet games
- Mobile games

**Hardware**

- Virtual reality
- Smartphone
- Equipment
- Console
- Tablet
- Joystick
- Mouse
- Device
- Screen

**Unclassified search terms**

- Machine learning
- Information technology
- Assistive technology
- Communication technology
- Inclusion
- Management
- Adapt*
- Support*

Following research recommendations for searching gray literature and the need to reduce issues associated with bias from software and algorithms and reproducibility of the results [33,34,35], the databases and search terms and techniques for gray literature were tested and refined for relevance to the research questions and the academic literature already gathered. In the initial gray literature search, we explored the website of the International Alliance of ALS-MND Associations and the websites of ALS and MND not-for-profit organizations in Australia and the United Kingdom (2 countries with English-speaking populations and similar governance systems). The research team also identified and searched the websites of game and disability not-for-profit organizations based on previous research and practice. The process identified 7 articles for consideration to be included in the review. The small number of articles resulted in a refinement of the gray literature search and a greater focus on “lived experience” of MND and games and the experiences of those with similar motor impairment and movement issues.

The revised gray literature search explored the websites of ALS and MND not-for-profit organizations, game and disability not-for-profit organizations, and communication and assistive technology not-for-profit organizations in Australia, the United States, the United Kingdom, Canada, and New Zealand. Countries were selected based on work being published in English and the initial gray literature search and evidence from academic literature suggesting areas where the most relevant research and practice were being conducted. Furthermore, based on the initial search, relevant technology industry databases were searched: Google (general and corporate), Xbox, Microsoft, Apple, Entertainment Software Association, NeuroNode, and Tobii. Research, policy, practice guidelines, evaluation, information, communication, and similar documents were scanned on each website for relevant work. Searching was restricted by a time limit of 1 hour per website in line with past scoping review work and for helping adhere to the project’s timeline and revised search focus. The process resulted in 206 gray literature documents to be considered for inclusion in the review.

Academic and gray literature excluded were work involving animals, conference abstracts, book reviews, and work not published in English.

### Stage 3: Academic and Gray Literature Selection

The results from searches of all databases were consolidated, and all duplicates were removed. Following consolidation, and due to the relatively large number of results and limitations on research team capacity, titles and abstracts were screened by BO, MH, and ND, and those not relevant were removed. Abstracts were assessed based on the inclusion and exclusion criteria. Literature that remained unclear as to whether it was eligible for inclusion was reviewed by BO, MH, and ND until agreement was achieved. Group disagreements were resolved by KH and MH. Full-text documents were gathered and read independently by BO, MH, and KH. Study selection was also documented using the PRISMA-ScR (Preferred Reporting Items for Systematic Reviews and Meta-Analyses extension for Scoping Reviews) guidelines [32], which were also used for gray literature.

### Stage 4: Charting the Data

Data from academic and gray literature were extracted using a combination of inductive and deductive analysis, including the use of a framework based on past research and that was reviewed and refined based on the research questions [28,36]. The framework was piloted with a sample of the included studies and further refined by BO and ND. The framework was used to collect data on article title, author names, journal, publication year, population, kinds of technology, game type, context, concept, method, outcomes, whether the game was fun and enjoyable, barriers and opportunities, key findings, and strengths and limitations. The framework was created in Microsoft Excel to document data extraction.

### Stage 5: Collating, Summarizing, and Reporting the Results

Extracted data from academic and gray literature were summarized descriptively and compared for similarities and differences in a table [31] and to provide an overview of the available evidence as a whole. In line with the research questions, we explored and described data from studies on the kinds of technology or approaches that helped or could help people with MND and those with similar forms of motor impairment and movement issues and potential factors that may be barriers to playing video games. A number of factors were reported that may help to make games easier to play. These included changes to interface technology (eg, brain-computer interfaces [BCIs], eye gaze trackers, touch screen sensitivity, game controllers, and on-screen cursors and graphic interfaces); greater involvement of players in game development and design; and more support for physical, social, and economic challenges to better participate in games.

## Results

### Summary of the Literature Search

**Figure 1:**
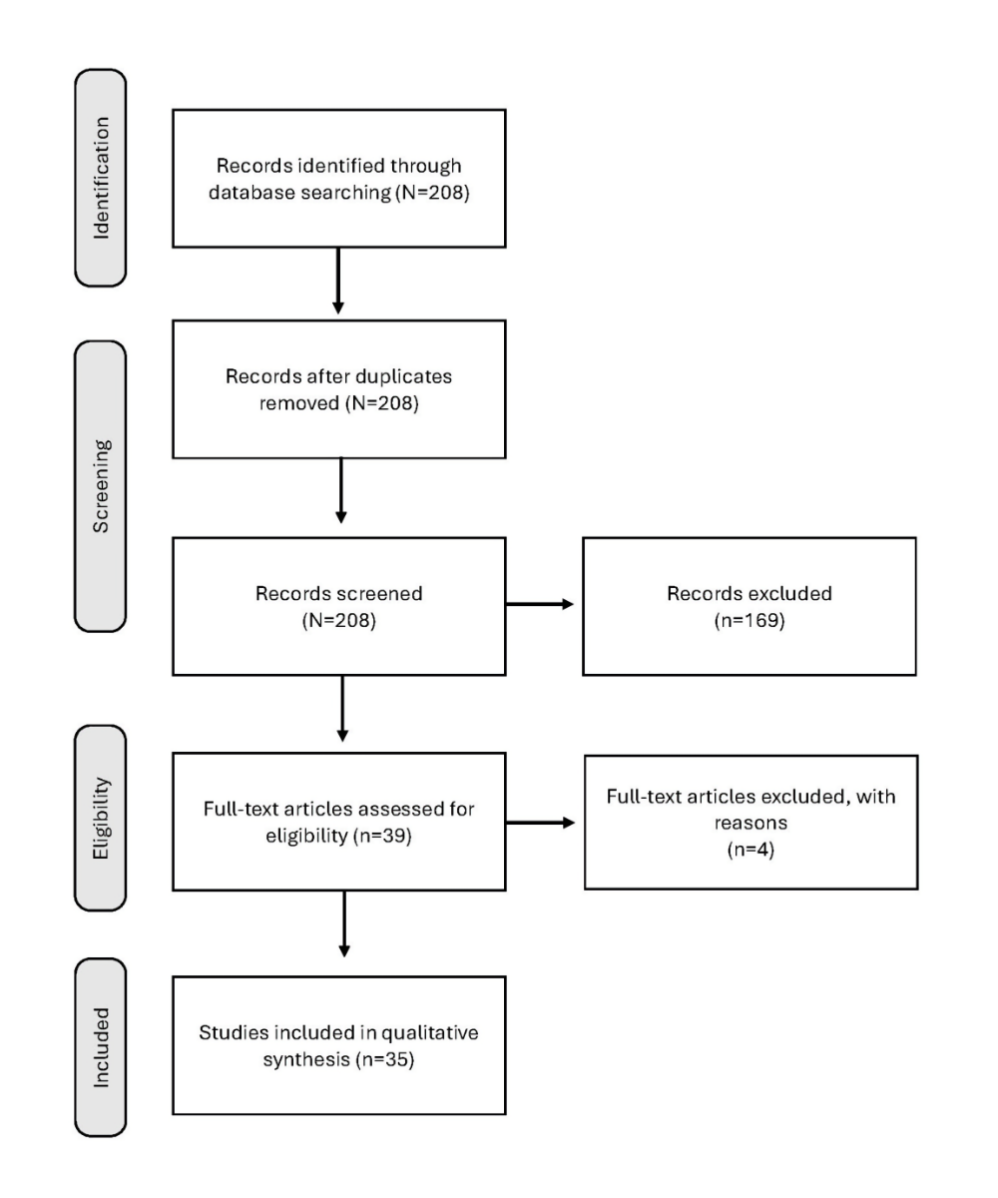
PRISMA-ScR (Preferred Reporting Items for Systematic Reviews and Meta-Analyses extension for Scoping Reviews) flow diagram [32].

**Figure 2:**
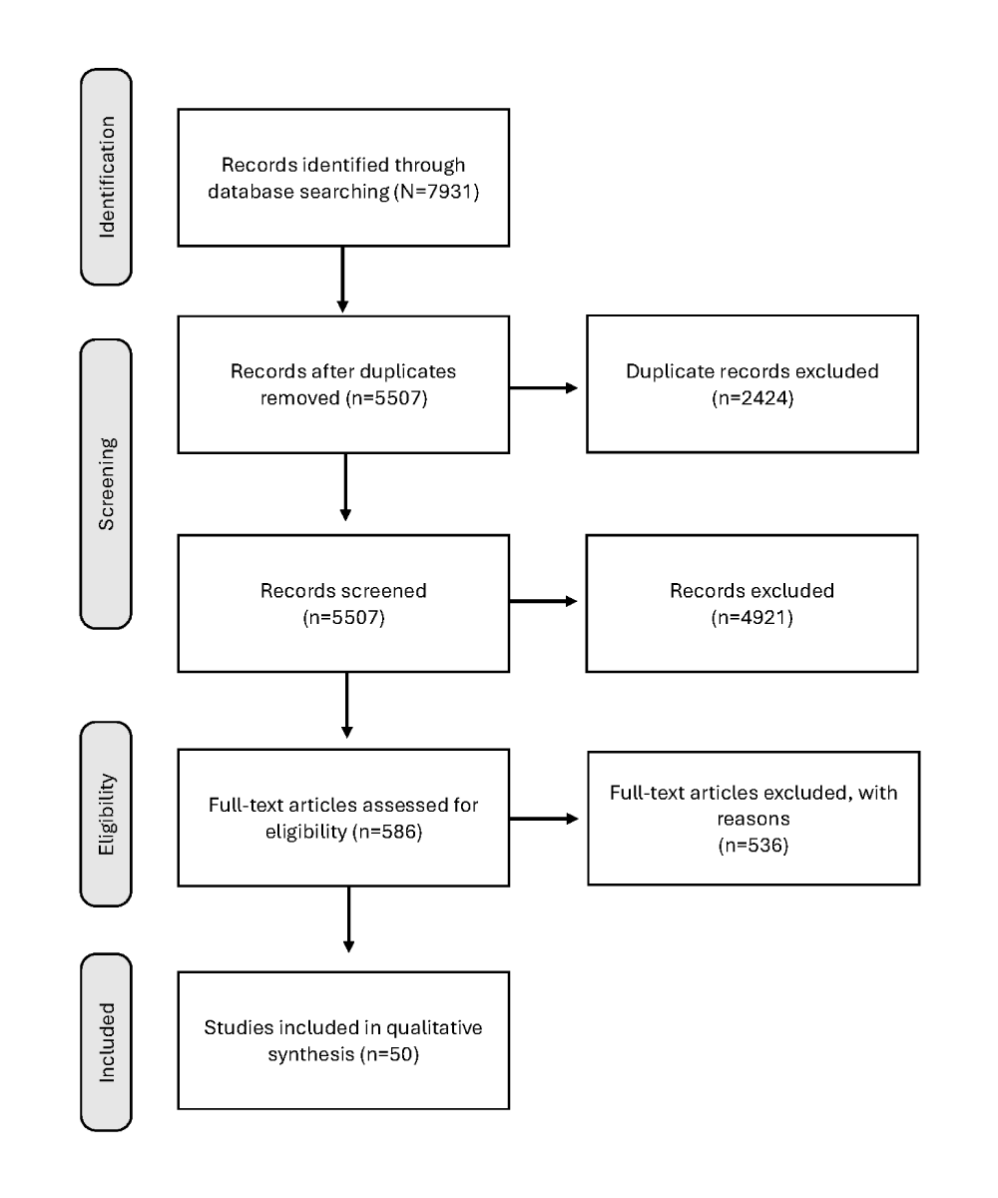
Gray literature search process flow diagram [33].

The search of academic literature found a total of 7931 records that were screened for eligibility, and 586 (7.39%) were selected for full-text assessment (Figure 1 [32]). As mentioned, the PRISMA-ScR instrument was used to filter articles that can be found in Multimedia Appendix 1. There were 208 records found in the search of gray literature, and 39 (18.8%) were selected for full-text assessment (Figure 2 [33]). A total of 85 documents were included in the review analysis (Table 1).

**Table 1:** Results of the literature scoping (N=85).

Category	Studies, n (%)	References	Summary description
Quantitative	35 (41)	Mackenzie et al [1] Lancioni et al [9] Nuyujukian et al [37] Uma et al [38] Holz et al [39] Scherer et al [40] Lancioni et al [41] Aliakbaryhosseinabadi et al [42] Cooper et al [43] Janthanasub and Ophapasai [44] Jaramillo-Gonzalez et al [45] Jarosiewicz et al [46,47] Kageyama et al [48] Käthner et al [49,50] Longatelli et al [51] Mott [52] Peters et al [53] Plotkin et al [54] Pousada et al [55] Sakamaki et al [56] Silversmith et al [57] Simeral et al [58] Simmatis et al [59] Sinha and Dasgupta [60] Sinha et al [61] Taherian et al [62] Isabela Lopes et al [63] Wallock and Cerny [64] Webb et al [65] Whittington et al [66] Wolpaw et al [67] Won et al [68] Zisk et al [69] Yao et al [70]	Research included studies of how effective information and communications technology was for better communication and control of computer and console systems for those with movement and motor impairment issues and focused on technology such as BCIs^a^; software used for software tools and objects; and hardware such as eye gaze trackers, robotic limbs, and headsets. Surveys were also conducted of experiences with and preferences regarding information and communications technology.
Lived experience stories, news, or information resources	20 (24)	AbleGamers [15] iPuntMidgets [18] Beemer [19] AbleGamers [71,72] Ryan [73] Barrett Centre for Technology Innovation [74] Delibasic [75] Gaddes [76,77] Neil Squire [78] Sharma et al [79] Reddit [80] Dall [81] Klassen [82] SpecialEffect [83] Apple Inc [84] Microsoft [85] AbleGamers [86,87] Bayliss [88]	Lived experience literature generally consisted of personal reflections, news, or reviews on gaming with disabilities; the limitations of controllers used to play games; and the accessibility features and related tools and software in games. Some of the literature discussed the gaming industry for more inclusive approaches to game development. Information resources mostly focused on game modifications for people with physical access needs.
Review	8 (9)	Pimentel-Ponce et al [89] Jamil et al [90] Kellmeyer et al [91] Pasqualotto et al [92] Linse et al [93] Neto et al [94] Pinto et al [95] McFarland and Wolpaw [96]	Studies included systematic and narrative reviews of research into communication and ALS^b^ and MND^c^, information and communications technology for people with disabilities, BCIs and video games, and the role of improved approaches to the use of technology for better communication and the ability to interact with software and hardware platforms. The literature also included assessments of evidence for technology prototype development.
Quantitative and qualitative	7 (8)	Hobson et al [36] Hernández et al [97] Holz et al [98] Martisius and Damasevicius [99] Pedrocchi et al [100] Karlsson et al [101] Thais Pousada et al [102] Metzler-Baddeley et al [103]	Research included literature exploring how effective information and communications technology was for better communication and control of computer and console systems for those with movement and motor impairment issues and experiences with and views on various interfaces, devices, and computer systems. Statistical analysis of technology use data, structured interviews, or participant commentary were methods reported in much of the literature.
Qualitative	6 (7)	Hellwig et al [104] Metzler-Baddeley et al [103] Dontje et al [105] Canella et al [106] Liberati et al [107] Versalovic et al [108] Judge et al [109]	The literature included reporting on focus groups, interviews, participatory design, and testing feedback to help explore the effectiveness of BCIs, games, and computer mouse control without the use of hands and communication changes for those with ALS and MND.
Guidelines	6 (7)	Microsoft [16] Game Accessibility Guidelines [21] Microsoft [22] Compañ-Rosique et al [110] Microsoft [111] Anhong et al [112]	The literature explored features and approaches to make it easier for individuals with disabilities to play video games on computers and consoles, including game industry developments.

^a^BCI: brain-computer interface.

^b^ALS: amyotrophic lateral sclerosis.

^c^MND: motor neuron disease.

### Key Findings

We analyzed the academic and gray literature (N=85) to describe the evidence base and identify relevant areas of research and practice for helping make video games more inclusive for people with MND. While there was a major gap in the literature on work involving people with MND and their experiences with video games, our analysis found several relevant areas.

The evidence base consisted of 6 categories: quantitative (35/85, 41%), quantitative and qualitative (8/85, 9%), and qualitative research (7/85, 8%); lived experience stories and information resources (21/85, 25%); reviews (8/85, 9%); and guidelines (6/85, 7%). Most of the academic literature explored customized information and communications technology for communication and control of computer systems (including desktop, laptop, smartphone, tablet, and console systems). In line with guidance on types and quality of evidence [113], the body of evidence in the academic literature was generally of a low level of quality. This was due to limitations in study design as differing types of technology were evaluated in a variety of ways and with differing sample sizes of participants, inconsistent results across the literature, and informal evidence on games. BCIs were the most common interface studied across all categories [37,39,40,42,46-50,53,57,58,62,67-70,90-92,98,99,107,108,114,115]. Only some of the documents in the academic literature focused on video games, with no or little involvement of people with MND [37,39,40,89,97,104,110]. Most of the studies on video games were of a low level of quality and a mixture of quantitative, qualitative, and review research.

Gray literature, including information resources, guidelines, and lived experience stories, generally focused on customized video game software (including accessibility features in games and gameplay) and hardware (including modified controllers and equipment) [15,16,18,19,21,22,71-88,111,112]. Most of the gray literature was of a very low quality due to significant limitations in study design and risk of bias, publication bias, and inconsistency of results. Much of the gray literature drew on anecdotal information and very small numbers of participants. One document of the academic literature on guidelines discussed user feedback and technology development to evaluate guideline recommendations, and this may be a useful example for future guideline work [110]. However, again, the document reported on evidence of a low quality.

Common factors were reported that suggest barriers and enablers or ways of use of video games or information and communications technology (Table 2). Common factors noted and that may function as barriers to enjoying video games were difficulties with game playability [15,16,21,22,71,72,85-88,104,110-112] and the physical requirements for interacting with games and their software and hardware [15,16,21,22,71,72,74,80-82,85-87,104,111]. Common factors reported that may be enablers for helping make games easier to play were changes to game software and hardware and game development to better meet the needs of players with disabilities [15,16,21,22,71,72,74,80-83,85-87,104,111,112]. Most documents about video games were gray literature of a very low evidence quality. The very low quality significantly reduces the reliability of the reported factors that may constitute barriers and enablers regarding making video games easier to play for those with MND. However, a major strength of the evidence was that it did include information not reported in the academic literature; drew on information in “real-world” contexts; and, critically, shared lived experience of video games and disability.

**Table 2:** Common factors reported that constitute potential barriers to and enablers of playing video games experienced by people with amyotrophic lateral sclerosis and motor neuron disease or others with similar motor impairment issues.

Technology and references		Potential barriers	Potential enablers
**Customized information and communications technology for communication and control of computer systems (categories: quantitative, quantitative and qualitative, qualitative, and review research)**			
	Nuyujukian et al [37] Uma et al [38] Holz et al [39] Scherer et al [40] Cooper et al [43] Janthanasub and Ophapasai [44] Jaramillo-Gonzalez et al [45] Jarosiewicz et al [46,47] Kageyama et al [48] Käthner et al [49,50] Longatelli et al [51] Mott [52] Peters et al [53] Plotkin et al [54] Pousada et al [55] Sakamaki et al [56] Silversmith et al [57] Simeral et al [58] Simatis et al [59] Taherian et al [62] Isabela Lopes et al [63] Wallock and Cerny [64] Whittington et al [66] Wolpaw et al [67] Won et al [68] Zisk et al [69] Yao et al [70] Jamil et al [90] Kellmeyer et al [91] Pasqualotto et al [92] Linse et al [93] Pinto et al [95] Holz et al [98] Martisius and Damasevicius [99] Pedrocchi et al [100] Karlsson et al [101] Thais Pousada et al [102] Metzler-Baddeley et al [103] Dontje et al [105] Canella et al [106] Liberati et al [107] Versalovic et al [108] McFarland and Wolpaw [96] Judge et al [109]	Varied or limited technology performance that limits ease of use, including time delays when typing or interacting with software and other difficulties using technology; lack of power; poor internet access; and weight, portability, or body fitting issues	Increased ability of the user to control and interact with hardware and software to undertake a range of daily life activities including communication
	Mackenzie et al [1] Lancioni et al [9] Hobson et al [36] Holz et al [39] Lancioni et al [41] Jaramillo-Gonzalez et al [45] Käthner et al [49] Longatelli et al [51] Plotkin et al [54] Pousada et al [55] Simmatis et al [59] Whittington et al [66] Wolpaw et al [67] Jamil et al [90] Pinto et al [95] Holz et al [98] Martišius and Damaševičius [99] Pedrocchi et al [100] Karlsson et al [101] Metzler-Baddeley et al [103] Canella et al [106] Liberati et al [107] Judge et al [109]	Difficulties using technology due to limited physical movement and strength, fatigue, poor vision, and other similar issues associated with disease or disability	Increased ability of the user to restore functionality for communication and control of hardware and software through more accessible and customized technology that meets their physical and related needs
	Mackenzie et al [1] Hobson et al [36] Peters et al [53] Pousada et al [55] Silversmith et al [57] Wolpaw et al [67] Sharma et al [79] Jamil et al [90] Kellmeyer et al [91] Neto et al [94] Pinto et al [95] Pousada et al [102] Metzler-Baddeley et al [103] Canella et al [106] Liberati et al [107] Versalovic et al [108] McFarland and Wolpaw [96] Judge et al [109]	Issues with affordability, availability, community awareness, skills to use technology, support from others, and ability of technology to meet changing abilities due to disease progression	Increased ability of the users to access and use a range of devices and technology that meet their needs with support from others in the community and professionals
**Video game software and hardware (categories: lived experience stories, news, or information resources; guidelines; quantitative and qualitative research; and qualitative research)**			
	AbleGamers [15,16] Game Accessibility Guidelines [21] Microsoft [22] Compañ-Rosique et al [110] Hellwig et al [104] AbleGamers [71,72] Microsoft [85] AbleGamers [86,87] Bayliss [88] Metzler-Baddeley et al [103] Microsoft [111] Anhong et al [112]	Difficulty with game playability, including difficulty level, speed, menus, graphical or hardware interfaces, a lack of alternatives for gameplay, a lack of engaging game design, and problems with software interfaces for providing feedback and social interactions	Increased ability of the user to control and interact with game software and enjoy gameplay more due to having multiple options for interfaces and interactions, including those sensitive to fatigue or movement and mobility difficulties, use of sliders and circular meters and devices that do not require the use of hands, or configurable speed or remappable controls
	AbleGamers [15,16] Game Accessibility Guidelines [21] Microsoft [22] Hellwig et al [104] AbleGamers [71,72] Barrett Centre for Technology Innovation [74] Reddit [80] Dall [81] Klassen [82] SpecialEffect [83] Microsoft [85] AbleGamers [86,87] Microsoft [111]	Difficulty with the physical requirements for interacting with games (including difficulty with console controllers and movement and mobility, such as limited hand or speech function)	Increased ability of the user to restore functionality for control of game hardware and software through more accessible and customized technology that meets their physical and related needs

Literature on information and communications technology suggested that factors that may be barriers to its use and access were varied or limited technology performance that negatively impacts ease of use [37-40,43-59,62-64,66-70,90-93,95,98-103,105,106,115-117]; difficulties using technology due to physical limitations associated with disease and disability [1,9,36,39,41,45,49,51,54,55,59,66,67,90,95,98-101,103,106,107,117]; and issues with affordability, availability, skills, awareness, and related challenges [1,36,53,55,57,67,79,90,91,94,95,102,106-108,115,117]. At the same time, the literature indicated factors in technology use that could potentially enhance a user’s capability of a user to access and use a range of devices and technology for communication, participating in daily life, and better meeting their needs (Table 2). Overall, this body of literature was of a low quality. Again, the low quality significantly reduces the reliability of the reported factors that may constitute barriers and enablers regarding the use of information and communications technology, including for playing video games. However, the literature had major strengths. Many individual documents showed strong study designs, were completed in line with the ethical requirements of various universities and similar institutions, conducted practical evaluations of technology to identify what may or may not help reduce the burden of disability in future work, and shared feedback from people with MND and similar conditions on their experiences with technology.

### Categories of Relevant Research and Practice: Detailed Description

#### Quantitative Research

The literature in the category of quantitative research focused on the experiences of people with ALS and MND and similar forms of movement and motor impairment issues when using desktop, laptop, smartphone, or tablet computer systems. Most of the documents in this group evaluated participant interaction with and control of the computer systems using customized interfaces or devices [1,9,37-69]. BCIs were the most common interface studied [37,39,40,42,46,47,49,50,53,57,58,62,67-70]. The literature on BCIs explored how participant brain activity could control on-screen keyboards, cursors, menus, and other software on computer systems. In total, 3 documents, which were about BCIs [37,39,40], and 1 document, which was about smart home technology [64], explored video games, but their focus was primarily on the effectiveness of the interface technology rather than on the games played. The remaining documents [38,41,43-45,51,52,54,56,59-61,63,64,66] evaluated vision or eye gaze tracker interfaces, smartphone or touch screen interfaces, robotics or passive and active physical supports, graphical or software interfaces, and auditory interfaces.

The second group of documents [1,9,41,48,55,65] in this category surveyed the experiences of people with ALS and MND and others with similar movement and motor impairment issues with a variety of interfaces and devices used for computer systems as part of daily life and in specific government-supported programs. One document also reported on the views of professionals supporting decision-making regarding technology. However, none of the literature in this group explored video games.

#### Lived Experience Stories, News, Reviews, and Information Resources

Literature in the category of lived experience stories, news, reviews, and information resources included sharing of insights, technology options, and advice for people with ALS and MND and similar forms of movement and motor impairment issues when using customized game software and hardware interfaces or accessibility features to play video games on console (eg, Microsoft, Nintendo, or Sony consoles), desktop, laptop, smartphone, or tablet computer systems. A large part of this group contained news on developments in the game industry and game communities for making games easier to play for those with disabilities [71-79]. The documents included news on special controllers; industry changes to help enable a better understanding of gaming for those with disabilities and relevant work; and different play styles and features in games, such as subtitles, remapping of buttons, and automating aspects of gameplay.

A smaller part of this group explored stories of lived experience with disability or ALS and MND and games [80-83]. Documents included reflections on overcoming challenges to enjoy playing games (eg, difficulty holding controllers and reaching buttons and game speed), playing games with family and friends, learning from others how to modify games or use customized software and hardware to play them, and the pure fun and joy of playing games. Another small part of this group provided information and advice on practical aspects of how to play games with a disability, including changing game software and making changes to rooms and equipment to help play games with changing abilities due to disease progression [15,83,84]. Finally, 3 documents were reviews of games or console hardware, with quality assessments for ease of playing with disabilities and what worked or did not work (Table 2) in gameplay more generally [86-88].

#### Reviews

A total of 3 documents in this category reviewed evidence of how effective BCIs were for communication and use of computer systems by people with ALS and MND or similar forms of movement and motor impairment issues [90-92]. BCIs enabled communication and use of computer systems, but there were limitations in their use. Similarly, another 3 documents found that a variety of interfaces, devices, and computer systems could enable communication and use of computer systems, including older and new and advanced forms of technology, but with limitations [93-95]. One document explored gamification and neurological motor rehabilitation [89]. While focused on rehabilitation, the document found that motion-controlled nonimmersive virtual reality could be used to interact with console systems. However, some consoles and games that were more costly or required greater user mobility could limit accessibility.

#### Quantitative and Qualitative Research

The largest group of literature in this category reported on the development and evaluation of interfaces and systems—a low-cost video game controller, a long-term independent BCI, a multimodal neuroprosthesis, and a BCI gaming system [97-100]. Study participants were able to use the technology for communication and to interact with computer systems, but there were limitations to this use. The remaining 3 documents in this category explored the experiences with and views on low-cost and do-it-yourself assistive technology; eye gaze tracking devices; and laptops, tablets, and other forms of digital technology of people with ALS and MND or similar forms of movement and motor impairment issues [36,100-102]. Technology was found to enable communication and use of computer systems, but again, there were challenges in its use.

#### Qualitative Research

A total of 4 documents in this category focused on the views and experiences of people with ALS and MND or similar forms of movement and motor impairment issues when using a variety of desktop, laptop, smartphone, or tablet computer systems and including a hands-free mouse [103,105,106,116]. A total of 2 documents in this group explored the views and experiences of people with ALS and MND regarding BCIs [107,108].

#### Guidelines

Most of the literature in this category addressed the need for more information and awareness of why there is a need to make video games more accessible for people with disabilities and how this can be achieved through technical development, organizational changes, and engagement with relevant stakeholders [16,21,22,110,111]. Documents included guidelines and advice for game developers and players. Guidelines, which were mostly developed by Microsoft [16,22,111], addressed a range of topics, including the social experience of gaming; practice frameworks for more accessible games (including development for motor, cognitive, vision, and other challenges); and features of technology, such as remappable controls, adjustable screen settings, game difficulty levels, and subtitles and prewritten communication options (eg, chat wheels or a “quick reply” option). One document explored potential areas of concern about the use of artificial intelligence technology and how it may impact people with disabilities without appropriate design, development, and testing [112].

## Discussion

### Principal Findings

Researchers have established that accessing the lived experience perspectives of people with MND is crucial for better understanding their needs for relief from symptoms of the disease, creating support programs for them, their family, and loved ones, focusing on enhancing communication and technology use. However, our review found that, problematically, there was very little study of the lived experiences of people with MND when playing video games, what can help make playing games easier, what sorts of social experiences with video games are preferred, and what can help with feelings of social connection. It is telling that 91% (77/85) of the academic and gray literature reviewed did not draw from actual lived experience of MND and playing video games. Due to this major gap in understanding, our discussion focuses on 5 priority areas to make adjustments for advancing future research and practice. The areas are the role of methodological issues in limiting the voices of people with MND; the importance of gray literature for balancing research; what may help involve more people with MND in video game research and development; how changes to program and policy work can support more affordable and available technology for playing games that meets changing abilities due to progression of MND; and potential improvements to gameplay features, game development guidelines, and game industry processes.

While the evidence is significantly limited, it is promising for future work because it does suggest that some players with MND have used interfaces to interact with consoles, computers, and other devices for playing games. The interfaces can help reduce problems with a lack of physical ability to communicate with and input information into a game, such as controlling cursors on menus or a character [15,38,114]. Interfaces include hardware such as keyboards, joysticks, mice, wearable sensors, and eye gaze tracking and software that helps change the sensitivity of devices and remap buttons and keys so that they are easier to press. Games played by people with MND also suggest the importance of certain game design principles that create rich yet accessible experiences, as well as their potential social benefits.

### Methodological Decisions Restricting the Voices of People Living With MND

The voices of those with lived experience have become a critical rallying cry for the disability rights movement as the framing regarding empowered decision-making has become “nothing about us without us” [11]. Previous research has also found that people living with MND are experts on their experience with the disease in addition to the health, technology, and other professionals who support them [118]. Meaningful engagement with people living with MND can yield important lessons for research and practice [119] and prioritize specific areas of focus for research to help improve outcomes and better meet their needs [120]. Unfortunately, the scoping of the academic literature highlighted that this sentiment has not yet translated to the research sphere. Reviewing the identified literature in the field of health was particularly emblematic of this dearth of voice, with studies focused on technical measurement or developing a theoretical proof of concept without considering the expertise of those whom the supports or interventions were intended to help.

While disappointing, it was more surprising that literature from the fields of the humanities or “soft sciences” provided barely more opportunities to understand the voices of people who are gamers and also living with MND. To borrow a quote from fictional chaotician Dr Ian Malcolm in the film *Jurassic Park*, “...your scientists were so preoccupied with whether or not they could, they didn’t stop to think if they should” [121]. When understanding the ways in which participation in gaming can improve quality of life for people living with MND, scientific inquiry is of course essential, but an expanded view of what constitutes “effective” needs to consider not just baseline and posttest results but also the ways in which supports and interventions are experienced by the participants and whether these concepts are fit and preferable for their everyday lives.

An abundance of literature reporting on BCIs brings to attention the value of lived experience (Table 1). While, on the surface, these technologies are impressive in their potential demonstrated in laboratory conditions, questions regarding transfer to everyday living and cost of access remain unanswered. Institutions that reward the registration and sale of patient subscriptions and data create a perverse incentive, presenting a significant ethical dilemma for those developing these technologies. If a technology can empower gameplay for the duration of the study but the individual cannot afford to access that technology once the study is completed, how does this really improve quality of life for the participant? Arguably, generating new knowledge for that person is only meaningful if there is a pathway for it to be translated beyond the laboratory to help either themselves or others at some point in the future. This raises serious ethical implications in terms of how higher education institutions and their commercial partners intend to monetize and gatekeep the technologies that have been tested through the participation of people living with MND.

Ethical considerations in academic research may be an important avenue for addressing methodological challenges in the types and quality of research on people living with MND and video games. The work reported in both academic and gray literature was overall of a low quality. To help improve the understanding and applicability of the research on the lived experience of MND and video games, drawing on the strengths of academic and gray literature and mitigating their limitations is a useful consideration for future work. First, while the gray literature was of a very low quality, it contained information on perspectives on gameplay, game culture, and game-based information and communications technology that was not well represented in the academic literature. The gray literature also reported on “real-world” contexts. These areas of focus could benefit the development of study designs in academic contexts, address significant gaps in the research, and help better understand relevant barriers and enablers regarding video games for players with MND in everyday life, as demonstrated by previous research on other forms of relevant technology and their use in community settings [122]. At the same time, the major challenge of the evidence in the gray literature was its very low quality, which reduced the reliability of its findings. The ethical processes and procedures of academic research, including informed consent, use of validated tools and approaches, and data confidentiality [123], can help in gathering more reliable evidence in ways that are sensitive to the needs of people with MND. Academic work can also support greater reach in studies and sharing of information on what helps those with MND enjoy video games through their networks and relationships with relevant stakeholders in the technology, community, health, and disability sectors.

### Your Princess Is in Another Castle: Finding Voice in Gray Literature

All is not lost as people living with MND and their allies have found other avenues to have their voices heard. While there was a paucity of academic literature that investigated the lived experiences of interfaces and programs of research that support people living with MND in playing video games, gray literature such as blogs by advocates or community-focused websites sought to address this oversight (Table 1). Whether it was nonprofit organizations such as AbleGamers or individuals who took it upon themselves to share their ideas and solutions, the research team found that lived experience of implementation in real-world contexts “filled in the gaps” that were evident in the academic literature. For example, little of the academic literature reported on the types of games that people living with MND actually wanted to play. Multiple research studies used simple games (eg, “tic-tac-toe” or “Connect4”), but the authors themselves often acknowledged that game presentation and game design were an afterthought or ignored completely as the purpose of using these games was to test the functionality of a new interface technology (Table 1) [39,40]. Gray literature authors discussed games such as *Monument Valley* and *Civilization VI* [124], detailing their enjoyment of the esthetics and more complex game mechanics. Both of these titles have been reviewed positively by the mainstream gaming press [125], highlighting that gamers living with MND want to access the same experiences as those of gamers without disabilities. The desire to access the same experiences as fellow gamers is echoed by innovative work in participatory design for gesture-based gaming with people with disabilities. Such work has already established that it is possible to draw on existing technology and work with users to create more inclusive, satisfactory, and entertaining gaming experiences [126].

More broadly at a methodological level, this scoping review highlights an affordance of including gray literature in any review that seeks to address interventions or supports for historically marginalized populations. Translating research into impact requires an awareness of context and contextual fit. In the gray literature identified through this scoping review, nonacademic perspectives served as a response to the lack of consideration given to research translation in academic literature. As an illustration of this value proposition, if a BCI or eye gaze device is going to cost an exorbitant amount for an individual, then surely such practical barriers for many people living with MND require urgent attention by those conducting the studies. Through this scoping review, the research team found that it was almost exclusively through the gray literature that these concerns were raised (Table 1). Clinical studies investigating proof-of-concept technologies are an important first step in showing what is possible, but consideration needs to be given to the likelihood of such technologies ever being within the affordability range of the participants. Future research and development must engage with issues of availability and potential barriers to technology and consider approaches such as rapid ideation. Rapid ideation can help widen the focus of research with participants to consider a wide range of technology devices that they may already access or use [127].

### Ways to Involve More People With MND in Video Game Research and Development

Learning from previous research on communication technology and MND provides important insights for researchers and practitioners for involving a greater and more diverse number of people with MND in video game research and development [1]. Nonprofit organizations that have existing relationships with people with MND and their family and loved ones, including organizations that help with education, information, access to information and communications technology, and support and services for health care and disability, can help with study design, awareness raising, recruitment, and accessible participation in ways that better meet the needs of people with MND [1,12]. Organizations focused on MND in particular, such as MND associations in the United Kingdom, Canada, and Australia, as well as assistive technology and MND clinics, are important given their involvement in previous research, existing protocols, and quality processes for participating in research and information-sharing activities. Collaborating with nonprofit agencies and clinics will help increase awareness of research and development projects and help with greater participation of people with MND. Collaboration can also improve insights across a range of settings, including medical, university and community contexts. An aim is to obtain a better understanding of what helps with the application of technology in everyday life.

Furthermore, engaging with the communication and other participation preferences of people with MND is also very important to better represent their voices and for more inclusive video game research and development. First, involving people with MND in the development of study designs, including their views and preferences on relevant areas to explore based on their lived experience of video games and how to conduct research, may benefit future studies and is in line with the evidence base [6,115]. Qualitative and quantitative research is required, but greater use of exploratory qualitative research and user-driven techniques in game development is needed given the significant lack of this work in the field. Second, considering specific issues related to MND and communication is likely to help. Attention must be paid to the need for flexibility in participation and multiple options for being involved to help reduce issues associated with fatigue, physical movement and mobility, and support for carers and health care and disability professionals [1,10]. Communication about research must also consider the variety of feelings and perceptions associated with the technology used to play video games and be tailored accordingly. For example, some people with MND may feel hesitation or concerns about what it means to use customized interfaces to play video games, whereas others have great interest and a sense of hope about the potential of finding it easier to play video games and spend time with others [1,108].

Recognizing that the expertise of people living with MND can extend to expressions of creativity for game development may also help with involving participants in research and practice. People living with MND are potentially a unique source of insights for developing different ways of using technology, content topics for video games, and the social channels supporting games beyond the more general need for inclusive games. For example, people with MND and their carers who participated in “photovoice” research were able to use digital cameras to take photographs of their daily lives and write descriptive text of the images [128]. Participants found that the experience led to discussion of difficult feelings and challenges but also resulted in feelings of hope and positivity and the ability to come up with creative solutions to accessing technology. While not game-based work, the aforementioned study suggests that involving people living with MND may help them to make a positive contribution beyond the stigma often associated with disease and disability. There are unexpected benefits from the adoption of inclusive games. The study also showed strong similarities with a culture of documenting and sharing experiences playing video games [129]. A greater number of people living with MND documenting their gameplay and approach to using technology may be able to broaden and expand this part of gaming culture.

### Advocating to the Government for Policy and Legislative Change

Building on the potential for game researchers and developers to collaborate with nonprofit organizations that support people living with MND, it is similarly possible to gather learning from this work for advocating to the government for policy and legislative change at national levels. A greater understanding of what may or may not work in research, programs, and activities on more inclusive games for people with MND can inform and shape advocacy to help achieve structural change through legislative reform. Advocacy to the government for improved access to more subsidized support for more relevant and a greater number of technology and recreational activities, such as through the National Disability Insurance Scheme in Australia, could help make video games more affordable to a greater number of people with MND. Researchers in Australia have called for changes to the ways in which people are assessed for eligibility to access the National Disability Insurance Scheme so that assessments are fairer and more inclusive [130,131], including for technology that can be used to play video games. Collaborating with other organizations advocating to the government for improved access to technology for people with disabilities and in a collective fashion may be strategic in working toward legislative change relevant to inclusive video games. People living with MND in New Zealand, Canada, the United Kingdom, and other countries with similar social safety nets may also benefit from similar forms of advocacy and legislative change for improved access to technology.

Higher-level advocacy can also create awareness of the need for funding to support training and other professional development activities involving MND community organizations, game and technology developers, and health care and disability professionals [1]. Advocating for and securing funding that supports a range of professionals, including health professionals, could better support MND clinics and their ability to refer patients interested in video games to appropriate assistance [132].

### Opportunities and Challenges for the Video Game Industry

In line with user-driven and participatory game design, there is a valuable opportunity to draw on the contributions of people with MND and game developers, including their creativity and innovation, to help enhance specific aspects of video gameplay and the guidelines and processes that shape development. Game industry professionals may benefit by considering how to make it easier for people with MND to perform gameplay interactions that are sensitive to fatigue or difficulties with movement and mobility, access multiple interface options sensitive to issues with fatigue or difficulties with movement and mobility, use sliders and circular meters and devices that do not require the use of hands, configure game speed, and remap controls. Companies can address options for easier gameplay for those with MND early in the game design and development process. It is possible to embed options in company policies and practice guidelines to shape the practical production of video games and gather feedback on the impacts of implementing specific changes throughout the concept development, game and technical design, art and sound development, production planning, and team management phases of game production [132]. The need for game design and development adherence to World Wide Web Consortium standards [133], diversity and inclusion policies [134], and broader industry recommendations on supporting accessibility and disability [21] are valuable opportunities to better support players with MND through existing operating frameworks in video game companies.

It is also important to consider the issue that newer titles do not necessarily automatically equate to a more accessible experience. While high-resolution sequels and remasters of classic video games may be more visually appealing to some players, the smaller graphical user interfaces found in many of these “improved” titles unintentionally make it more difficult for people who use information and communications technology to select options and press buttons [135]. The difference in user interface between the classic space empire simulation in *Master of Orion II* [136] and its later sequel perfectly exemplifies this issue and is an important reminder that accessibility needs to be a central consideration from the initial conceptualization of a game [137].

Moving beyond reductionist and “ableist” understandings of accessibility in video games [138], whereby the use of accessibility features is considered an “add-on” and not fully integrated into game design from the beginning [139], people with MND and game developers working together can create rich and engaging video games enjoyed by a range of people. Socially inclusive and high-quality video game experiences are not mutually exclusive. Indeed, trends in game development, including recent games such as *Diablo IV* [140], *The Last of Us* [141], *Return to Monkey Island* [142], *HyperDot* [26], and others suggest that well-received, commercially viable, and more inclusive games are not only possible but successful and important creative works [113]. Finding ways to expand recent approaches in game development is a unique opportunity to better meet the needs of people with MND in pleasurable, creative, and inclusive ways.

### Strengths and Limitations of This Scoping Review

There is no other study that exists on ways of making video games more inclusive for people with MND, and this is a major strength of our review. The peer-reviewed literature that we included drew on evidence-based approaches to exploring the experiences of those with MND or similar forms of motor impairment, video games, and information and communications technology. The gray literature identified several relevant areas of research and practice to help balance gaps in the peer-reviewed literature. As a result, both the academic and gray literature helped scope and identify the most relevant areas for future research and practice for making games easier to play for people with MND. Critically, this review also identified examples of the lived experience of MND and video games to help address the lack of lived experience with MND in research and ways in which this can be improved.

There was a major lack of evidence on the experiences of people with MND with video games, and this was an important limitation of this review. There is a risk that some relevant research was not included in this review. Furthermore, the lack of evidence significantly limited an in-depth understanding of what may or may not help make games more inclusive for players with MND. Small sample sizes in the studies also limited generalizability to a full and diverse range of people with MND. However, we mitigated the risks of a lack of evidence by developing a search strategy in line with recommendations for scoping reviews and our scoping review protocol. All evidence was assessed based on previous and relevant recommendations for the conduct of scoping reviews. Scoping reviews are also an established approach for finding the most relevant evidence on a topic where there has been little previous research. As such, this review identified the most relevant academic and gray literature available. Evidence from the literature provided valid options for future research and practice.

Finally, this review included gray literature. Researchers have noted that methodological quality, errors in reporting, and other limitations of gray literature can compromise the reliability of its findings and recommendations. However, in line with previous studies, and due to the lack of published work on the topic, the gray literature helped point out potential areas for future work that can address significant gaps in understanding. Gray literature has the potential to address research gaps, suggest new areas of concern in the field and mitigate potential problems.

### Conclusions

In general, our review of academic and gray literature exploring what changes to communication technology to improve social opportunities to play games found that, while it is an emerging field of research, there is a strong desire to better meet the needs of people with MND through video games that are more fun and easier to play. More inclusive video games and opportunities to enjoy them with others can make a significant and positive improvement in quality of life for those with MND. However, as our review found, there is a critical need for a comprehensive understanding of the experiences of a greater number of people with MND with playing video games and their relevant social experiences. Future work in a range of research, program, policy, and development activities must find ways to better include the views and preferences of people with MND regarding playing video games and make participation easier and more enjoyable.

## Appendix

Multimedia Appendix 1 PRISMA (Preferred Reporting Items for Systematic Reviews and Meta-Analyses) checklist.

## Abbreviations

ALS: amyotrophic lateral sclerosis
BCI: brain-computer interface
MND: motor neuron disease
PRISMA-ScR: Preferred Reporting Items for Systematic Reviews and Meta-Analyses extension for Scoping Reviews
