# Supplementary figures and images for: Making Video Games More Inclusive for People Living With Motor Neuron Disease: Scoping Review
Source: JMIR Rehabil Assist Technol. 2024 Dec 23;11:e58828. doi: 10.2196/58828 (PMC11704651; doi:10.2196/58828)

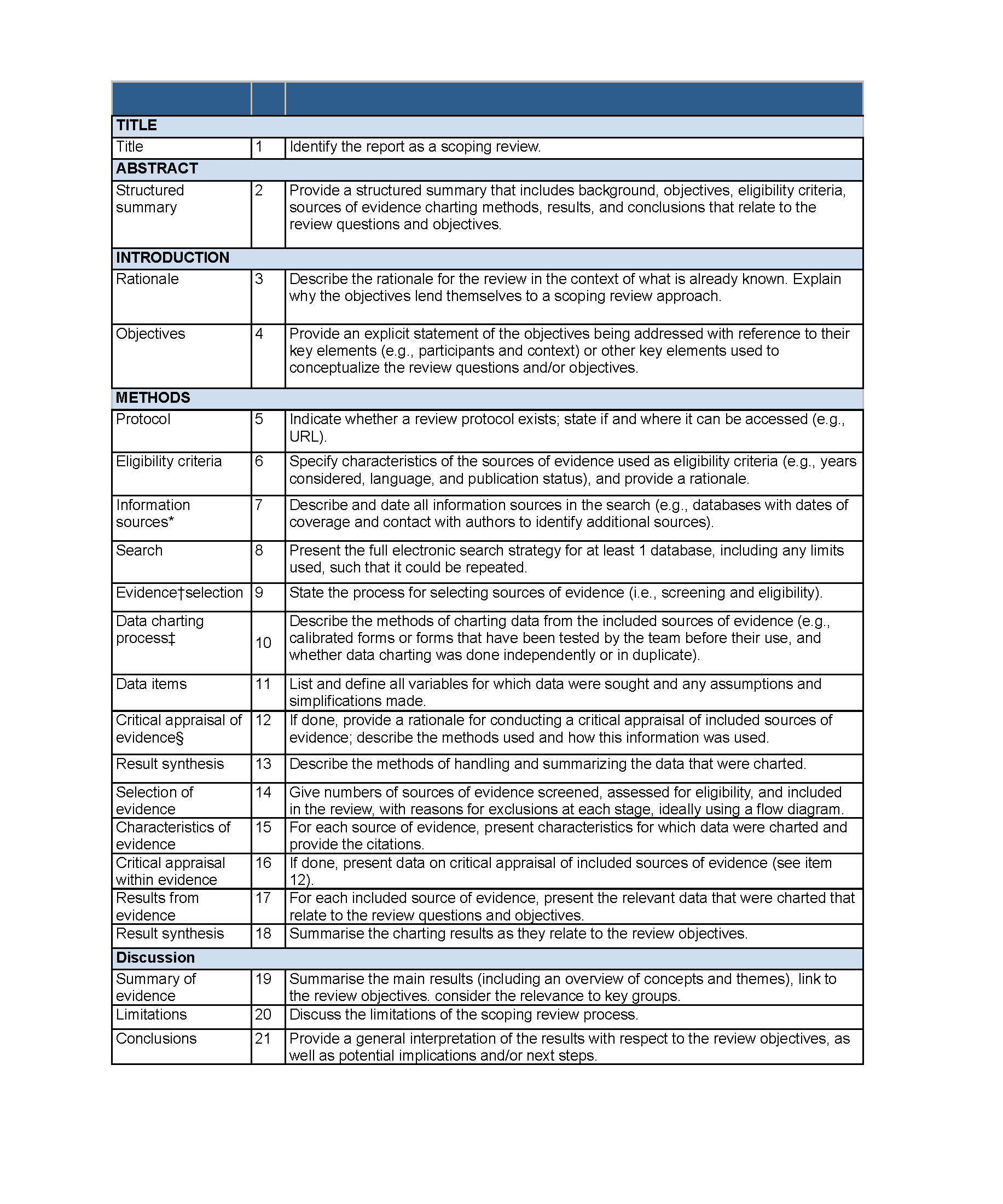

Supplement: Multimedia Appendix 1 [file rehab_v11i1e58828_app1.png]
